# Kidney-specific HIF-1α-dependent *ARL10*/miR-1271-5p overexpression in clear cell renal cell carcinoma

**DOI:** 10.1038/s41416-026-03399-w

**Published:** 2026-04-17

**Authors:** Patric M. Page, Tania Laperrière, Sonia A. Dastous, Sophie E. Landry, Patrick O. Richard, Michel Pavic, Sandra Turcotte

**Affiliations:** 1 Department of Chemistry and Biochemistry, Université de Moncton, Moncton, NB Canada; 2 Atlantic Cancer Research Institute, Moncton, NB Canada; 3 Department of Urology, Centre Hospitalier Universitaire de Sherbrooke, Sherbrooke, QC Canada; 4 Institut de recherche sur le cancer de l’Université de Sherbrooke, Sherbrooke, QC Canada; 5 Department of Hemato-Oncology, Centre Hospitalier Universitaire de Sherbrooke, Sherbrooke, QC Canada

**Keywords:** Renal cell carcinoma, miRNAs

## Abstract

**Background:**

Clear cell renal cell carcinoma (ccRCC) is primarily driven by chromosome 3p loss and inactivation of the von Hippel-Lindau (*VHL*) gene, which is frequently accompanied by chromosome 5q gain. However, the oncogenic contribution of 5q remains unclear. This study examines how 5q gain affects the expression of microRNAs implicated in ccRCC.

**Methods:**

Bioinformatic analyses were conducted to evaluate miRNAs associated with 5q gain and 3p loss. RT-qPCR validation was performed in tumour tissues and patient plasma. Mechanistic investigations integrated open-source ChIP-seq datasets and luciferase reporter assays.

**Results:**

miR-1271-5p overexpression was significantly associated with both 5q gain and 3p loss and strongly correlated with its host gene, ARL10. RT-qPCR confirmed elevated levels of miR-1271-5p and *ARL10* in ccRCC tumours and increased circulating miR-1271-5p in patient plasma. Mechanistically, these upregulations resulted from *VHL* loss and subsequent HIFα stabilisation. ChIP-seq datasets and luciferase assays demonstrated that HIF-1α, but not HIF-2α, directly binds within the intragenic region of *ARL10*. Importantly, this regulatory mechanism was specific to kidney cells.

**Conclusions:**

Coordinated upregulation of miR-1271-5p and *ARL10* reflects key genomic events in ccRCC and is driven by kidney-specific HIF-1a activity. Our findings suggest their promise for early detection and disease monitoring.

## Introduction

The survival outcomes for patients with clear cell renal cell carcinoma (ccRCC) depend on the disease stage at diagnosis. The 5-year survival rate for localised tumours (Stage I) is about 93.7%, but drops to 19.1% for metastatic ccRCC (Stage IV) [1]. Surgery with curative intent is primary for operable regional tumours, while targeted therapies and immunotherapies are used for inoperable and metastatic ccRCC [2, 3]. Despite advances in these therapies over the past decade, there is no curative treatment for advanced ccRCC [4]. Classic symptoms such as flank pain, haematuria, and palpable abdominal mass appear only in late stages [5]. Nearly 60% of patients are diagnosed incidentally, underscoring the need for new diagnostic tools for early detection [6].

Fortunately, large-scale genomic studies have mapped out the genomic landscape of ccRCC tumours. The evolution follows a branching trajectory shaped by key driver mutations [7]. Such affected genes include polybromo 1 (*PBRM1*), SET domain-containing 2 (*SETD2*), BRCA1-associated deubiquitinase 1 (*BAP1*), lysine demethylase 5C (*KDM5C*), and mechanistic target of rapamycin kinase (*mTOR*) [8]. Although 75% of identified driver events are subclonal, which increases intra-tumour heterogeneity, each clone is consistently preceded by two critical truncal events: (1) the loss of one copy of chromosome 3p and (2) the inactivation of the tumour suppressor von Hippel-Lindau (*VHL*) [9, 10]. These two events occur in more than 90% of patients with ccRCC [8, 11–13]. The VHL protein is part of an E3 ubiquitin-protein ligase complex that targets the oxygen-dependent degradation of Hypoxia-Inducible Factors (HIF-1α, HIF-2α) [14, 15]. In oxygen-deprived cells, HIFα is stabilised and binds to the constitutively expressed HIF-1β [16]. This transcription factor then binds to Hypoxia-Response Elements (HRE), activating the expression of hundreds of genes involved in pathways such as angiogenesis, glycolysis, and cell cycle regulation [17, 18]. Therefore, uncontrolled activation of HIFα in the absence of VHL promotes a constant pseudo-hypoxic environment suitable for tumour development [19, 20]. The loss of heterozygosity on 3p further corroborates the development of ccRCC, as it affects key local tumour suppressor genes such as *VHL*, *BAP1*, *PBRM1*, and *SETD2*. Usually, the loss of 3p results from an unbalanced translocation with chromosome 5q [21]. The concurrent gain of 5q may also impact tumour growth by amplifying proto-oncogenes; however, further research is necessary, as currently, only a few potential targets have been identified [22–24].

To explore this idea, we focused on the family of microRNAs (miRNAs). MiRNAs are short non-coding RNAs of approximately 20 nucleotides capable of negatively regulating gene expression [25]. Mature miRNAs bind to the argonaute (AGO) protein to form the highly stable RNA-Induced Silencing Complex (RISC) [26]. Dysregulation in miRNA expression has already been demonstrated to be involved in various cancers, including ccRCC [27–30]. Furthermore, their altered expression in disease-afflicted cells, such as cancer cells, can be detected in bodily fluids [31]. Assessing the expression of specific miRNAs in plasma and urine samples provides a new, simple, and cost-effective way to identify non-invasive biomarkers [32].

Therefore, to enhance our understanding of the potential oncogenic role of the lesser-known gain of chromosome 5q, we investigate the expression of localised miRNAs in ccRCC tumours. Among all miRNAs located on 5q, our analysis funnelled on miR-1271-5p, a miRNA that is overexpressed not only in tumours but also in plasma samples from ccRCC patients. The expression of miR-1271-5p significantly correlated with its host gene, ADP-ribosylation factor (ARF) Like GTPase 10 (*ARL10*), and both increased with 5q gain and 3p loss. Moreover, we observed that *ARL10* and miR-1271-5p overexpression were dependent on VHL loss and the subsequent increase in HIF-1α. Further validation with existing chromatin immunoprecipitation sequencing (ChIP-seq) data and luciferase reporter assays confirmed an active HRE in the intronic region of *ARL10*, close to the miR-1271-5p genomic site. Interestingly, a PanCancer analysis approach revealed that the promoting effect of HIF-1α was specific to kidney cells. Together, this research demonstrates that increases in miR-1271-5p and *ARL10* correlate with truncal events in ccRCC, further motivating their inclusion as early potential diagnostic tools.

## Methods

### Data acquisition and analysis

RNAseq and miRNAseq count data from The Cancer Genome Atlas (TCGA) PanCancer were downloaded from the GDC Data Portal (National Cancer Institute) and were processed with the EdgeR package (Version 4.4.2) [33]. Counts were normalised using the Trimmed Mean of *M* values. Differential expression analysis was conducted using the generalised linear model likelihood ratio test, considering miRNAs and genes significant when the absolute Fold Change (FC) was ≥1.5, and the False Discovery Rate (FDR) was <0.05. Arm-level copy number of Chromosome 3p, 5q, and somatic copy number data for *ARL10* and *HIF1A* from ccRCC patients were downloaded from http://firebrowse.org on October 14, 2025 (Broad Institute of MIT and Harvard) (Supplementary Tables 1–3). A chromosome arm was considered lost or gained when the GISTIC broad value was <−0.1 or >0.1, respectively. Similar copy number data from Renal Cell Carcinoma cell lines (*N* = 54) was downloaded from https://depmap.org/portal on January 22, 2025 (24Q4). For graphical representation, gene and miRNA expression are shown as Log Counts Per Million (LogCPM). HIF-1α, HIF-2α, and HIF-1β ChIP-seq data were acquired directly from the Gene Expression Omnibus (NCBI) (GSE200205, GSE120887, GSE130989). The sample acquisition process and the data analysis have been thoroughly described in the related papers [34–36]. Data analysis and visualisation were performed with the GVIZ package (Version 1.50.0) [37]. All analyses and graphical figures were generated with R software (Version 4.4.3) or GraphPad Prism 9. Kaplan–Meier survival plots were generated from https://kmplot.com/analysis/ on November 10, 2025 [38]. The median expression of miR-1271-5p and ARL10 was used as a cutoff.

### Clinical samples

Samples were collected from patients undergoing nephrectomy (*N* = 20) and from another cohort of metastatic ccRCC patients (*N* = 20) in accordance with the ethical protocol for research #2021-4061. Blood was collected in K2EDTA tubes, centrifuged at 1500 × *g* for 15 min, then again at 2500 × *g* for 15 min, to obtain plasma, which was stored at −80 °C. Plasma from healthy controls (8 men, 4 women over 50) was purchased from Innovative Research. Tumour and normal kidney tissue samples (~1 cm³) were stored in RNAlater at −80 °C.

### RNA extraction

Total RNA was isolated from cultured cells using TRIzol reagent (Invitrogen) combined with the Monarch Miniprep Total RNA extraction kit (New England BioLabs (NEB)), following the manufacturer’s instructions. Clinical samples were disrupted in TRIzol with a handheld homogeniser (SCILOGEX D160) and processed as previously described [30]. RNA concentration and quality were assessed using a NanoDrop 1000 spectrophotometer (Thermo Scientific). For plasma samples, RNA from 200 µL was isolated using the miRNeasy Serum/Plasma Advanced Kit (Qiagen) with the Cel-miR-39-3p spike-in (Qiagen) as a loading control.

### Quantification by RT-qPCR

For gene expression, 1 µg of total RNA underwent reverse transcription with SuperScript^TM^ III Reverse Transcriptase (Invitrogen) and oligo dT (20-mer) primers (IDT). Expression was quantified with SYBR^TM^ Green PCR Master Mix (Applied Biosystem) and primers (Supplementary Table 4) on a CFX Connect PCR machine (Bio-Rad). For miRNA, 100 ng of total RNA was reverse transcribed with MultiScribe reverse transcriptase and TaqMan^TM^ probes (Applied Biosystems, Supplementary Table 4). For plasma samples, 5 µL of RNA was used directly and normalised to Cel-miR-39-3p. Expression was measured with TaqMan^TM^ Universal Master Mix II with UNG and probes (Applied Biosystems). Gene and miRNA fold changes were calculated using the delta-delta Cq method, normalised with RPLPO or HPRT1 (gene) and RNU44, RNU48, or Cel-miR-39-3p (miRNA).

### Cell culture

Human RCC4 cells and their VHL-expressing counterparts (RCC4 VHL) were obtained from Millipore Sigma, while RCC10, 786-0, RCC10 VHL, and 786-0 VHL were generously provided by Amato J. Giaccia (Stanford University, CA). The A498 cell line was a gift from Réjean Lapointe (CRCHUM, Montreal, QC). The 769-P and RPTEC cell lines were acquired directly from ATCC. The A549, HCT-116, PC3, and T47D cell lines were kindly supplied by the Atlantic Cancer Research Institute (Moncton, NB). Details regarding the culture media used for each cell line are described in Supplementary Table 5. Cells were incubated at 37 °C in a humidified incubator with 5% CO_2_. To induce hypoxia, cells were placed in a H35 hypoxystation (Don Whitley Scientific) under 0.5% O_2_ and 5% CO_2_ for 16 h.

### Protein extraction and Western blot analysis

Proteins were extracted using M-PER lysis buffer as previously described [30]. For hypoxic experiments, UREA lysis buffer (8 M Urea, 150 mM β-Mercaptoethanol, 75 mM TRIS (HCl), pH 7.5), supplemented with protease and phosphatase inhibitors, and Benzonase nuclease (Sigma), was used instead. Protein concentration was measured with Pierce™ BCA Protein Assay Kits (Thermo Scientific^TM^) or Bradford protein assay (Bio-Rad), respectively. About 10 µg of protein was separated on SDS-PAGE gels and transferred onto a 0.45 µm PVDF membrane (Millipore Sigma). Membranes were blocked in 5% dehydrated milk, 0.075% Tween 20 (VWR) in PBS, then incubated overnight with primary antibodies against VHL (#68547), HIF-1α (#14179) or HIF-2α (#59973) (Cell Signaling) or β-actin (#sc-47778, Santa Cruz Biotechnologies). Afterwards, immunoblots were washed and incubated with HRP-conjugated secondary antibodies (Jackson ImmunoResearch). Protein visualisation was achieved on a ChemiDoc MP Imaging system (Bio-Rad) using ECL Prime Detection Reagent (Cytiva). For total protein staining, membranes were incubated in GelCode™ Blue Safe Protein Stain (Thermo Scientific).

### Luciferase reporter assay

The pGL4.R _TRE_minPro plasmid, gifted from Justin English (Addgene plasmid # 211517) [39], was digested using MluI-HF and HindIII-HF restriction enzymes (NEB). Genomic DNA from RCC10 cells was extracted using the Monarch Genomic DNA Purification Kit (NEB). Regions of interest were amplified by PCR using Q5 High-Fidelity DNA Polymerase (NEB) and primers (Supplementary Table 6). DNA amplicons were cloned into the open plasmid using the NEBuilder HiFi DNA Assembly Cloning Kit (NEB). Vector sequences were validated at The Centre for Applied Genomics (SickKids, Toronto, ON).

For the luciferase reporter assay, 5000 cells were plated in duplicates in a 96-well plate (Costar). After 24 h, 20 ng of each plasmid was transfected with DharmaFECT kb DNA transfection reagent (Horizon Discovery). The following day, Firefly luciferase intensity was measured using the luciferase Assay System (Promega) on a BioTek Synergy H1 (Agilent). Data were presented as the relative Firefly luciferase intensity compared to the original empty vector. To induce HIF in RCC4 VHL, cells were treated with 1 mM of Dimethyloxalylglycine (DMOG) for 24 h.

### Statistical analysis

Statistical analysis was performed using GraphPad Prism 9 or R (Version 4.4.3). Data is expressed as the mean ± standard error of the mean (SEM) of at least 3 independent biological replicates. All statistical tests used are described in the appropriate figure legends. Statistical significance was achieved when the calculated *P* value was less than 0.05.

### Ethics approval and consent to participate

The ethical protocol for research #2021-4061 has been approved by the Centre intégré universitaire de santé et des services sociaux de l’Estrie-Centre Hospitalier Universitaire Sherbrooke (CHUS) and the Université de Moncton for the acquisition and analysis of clinical samples. All methods were performed in accordance with the relevant guidelines and regulations. Informed consent was obtained from all ccRCC patients at CHUS.

## Results

### MiR-1271-5p and ARL10 overexpression in ccRCC correlates with classic genomic alterations

**Fig. 1:**
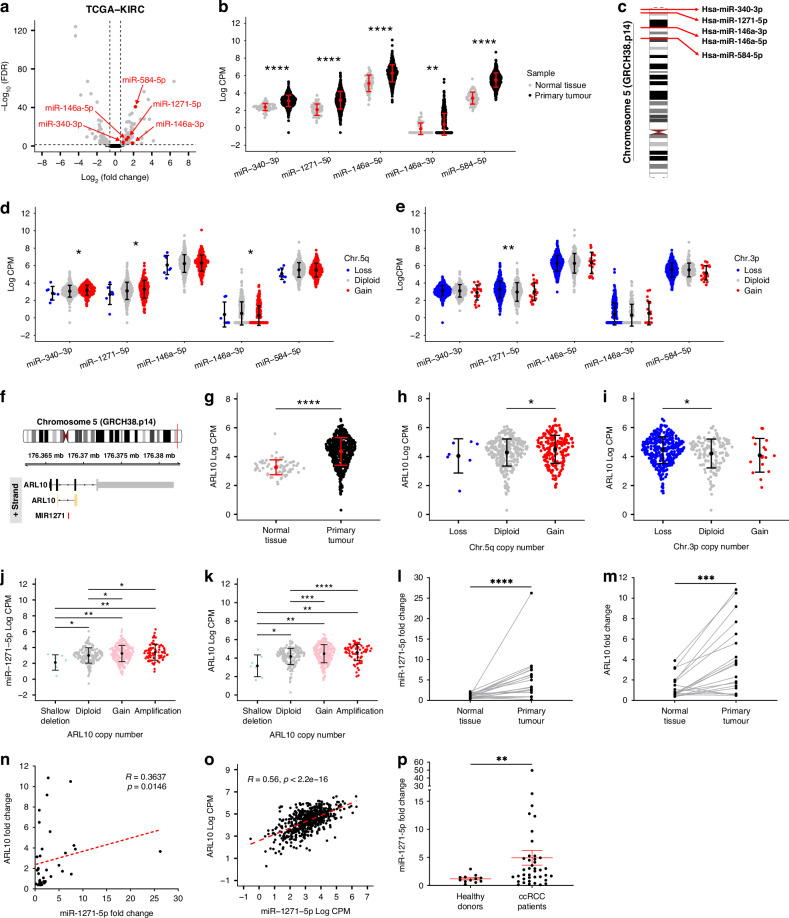
MiR-1271-5p and *ARL10* overexpression in ccRCC correlates with classic genomic alterations. **a** Volcano plot summarising the expression profiles of miRNA isoforms between tumour samples (*N* = 524) and normal kidney tissue (*N* = 69) from ccRCC patients (TCGA-KIRC). MiRNAs with an absolute FC ≥ 1.5 and an FDR ≤ 0.05 were considered significant (marked in grey). Significant 5q miRNAs are labelled in red. **b** Expression of significant 5q miRNAs in tumour samples compared to normal kidney tissue. **c** Genomic location of the significant 5q miRNAs. Expression of significant 5q miRNA in tumour samples based on arm level of 5q (**d**) or 3p (**e**) genotype. **f** Genomic location of miR-1271-5p and *ARL10*. **g** Expression of *ARL10* in tumour samples (*N* = 519) compared to normal kidney tissue (*N* = 70) (TCGA-KIRC). Expression of *ARL10* in tumour samples based on arm level of 5q (**h**) or 3p (**i**) genotype. Expression of miR-1271-5p (**j**) and *ARL10* (**k**) in ccRCC tumours based on *ARL10* copy number genotype. RT-qPCR quantification of miR-1271-5p (**l**) and *ARL10* (**m**) in primary tumours compared to paired healthy adjacent kidney tissue from consented ccRCC patients (*N* = 20). Spearman correlation between the expression of miR-1271-5p and *ARL10* in consented ccRCC patients (**n**) and the TCGA-KIRC cohort (**o**). **p** RT-qPCR quantification of miR-1271-5p in plasma from ccRCC patients (*N* = 40) compared to plasma from healthy individuals (*N* = 12). MiRNA expression was normalised with RNU48 (**l**) or Cel-miR-39-3p (**p**) while gene expression was normalised with HPRT1 + RPLPO (**m**). The data are presented as the Mean ± SEM. Statistical analysis was performed using Wilcoxon signed-rank tests (**b**, **d**, **e**, **g**–**k**), Wilcoxon matched-pairs signed-rank test (**l**, **m**), Mann–Whitney *U* tests (**p**), or Spearman correlation test (**n**, **o**). (*<0.05, **<0.01, ***<0.001, ****<0.0001).

Analysis of TCGA-KIRC data revealed 186 differentially expressed miRNAs with 77 downregulated and 109 upregulated in ccRCC tumours compared to normal kidney tissues (Fig. 1a). Of these significant miRNAs, only 5 (miR-340-3p, miR-1271-5p, miR-146a-5p, miR-146a-3p, miR-584-5p) are located on chromosome 5q (Fig. 1a–c). Comparing tumours with 5q gain (*N* = 210, 39.8%) to those with a diploid (*N* = 311, 58.9%) or deletion (*N* = 7, 1.3%) genotype, only miR-340-3p and miR-1271-5p exhibited higher expression. Conversely, miR-146a-3p expression decreased with 5q gain (Fig. 1d). Since 3p loss is a hallmark event in ccRCC and is frequently associated with 5q gain, we compared miRNA expression to the arm-level status of 3p. Tumours with 3p loss (*N* = 363, 68.8%) exhibited significantly higher levels of miR-1271-5p compared to unaltered tumours (*N* = 148, 28.0%) (Fig. 1e). Based on this observation, we focused our subsequent analyses on miR-1271-5p.

MiR-1271-5p is located in the second intron of the *ARL10* gene (Fig. 1f). Since intronic miRNAs are frequently co-expressed with their host gene, we simultaneously evaluated its expression. Similarly, ccRCC tumours express significantly higher levels of *ARL10* compared to normal kidney tissue (Fig. 1g). Additionally, tumours with either a gain of 5q or a loss of 3p express significantly more *ARL10* than diploid tumours (Fig. 1h, i). We further examine the impact of 5q gain by analysing *ARL10* somatic copy number, which revealed that most patients harboured either a gain (*N* = 250, 47.3%) or amplification (*N* = 81, 15.3%) of *ARL10* compared to diploid (*N* = 190, 36.0%) or decreased (*N* = 7, 1.3%) genotypes. Concordantly, expression of miR-1271-5p and *ARL10* both positively correlated with *ARL10* copy number (Fig. 1j, k).

Finally, we confirmed that miR-1271-5p and *ARL10* are overexpressed in tumour tissues from ccRCC patients compared to healthy adjacent kidney tissues (Fig. 1l, m, Supplementary Table 7). The expression of miR-1271-5p significantly correlated with ARL10 expression in our cohort and in TCGA-KIRC (Fig. 1n, o). Moreover, plasma levels of miR-1271-5p were significantly higher in ccRCC patients compared to healthy individuals (Fig. 1p). Taken together, these results suggest that 5q gain and 3p loss, often associated with ccRCC initiation, may contribute to the concurrent overexpression of local miR-1271-5p and *ARL10*.

### Expression of miR-1271-5p and *ARL10* among common cancer types

**Fig. 2:**
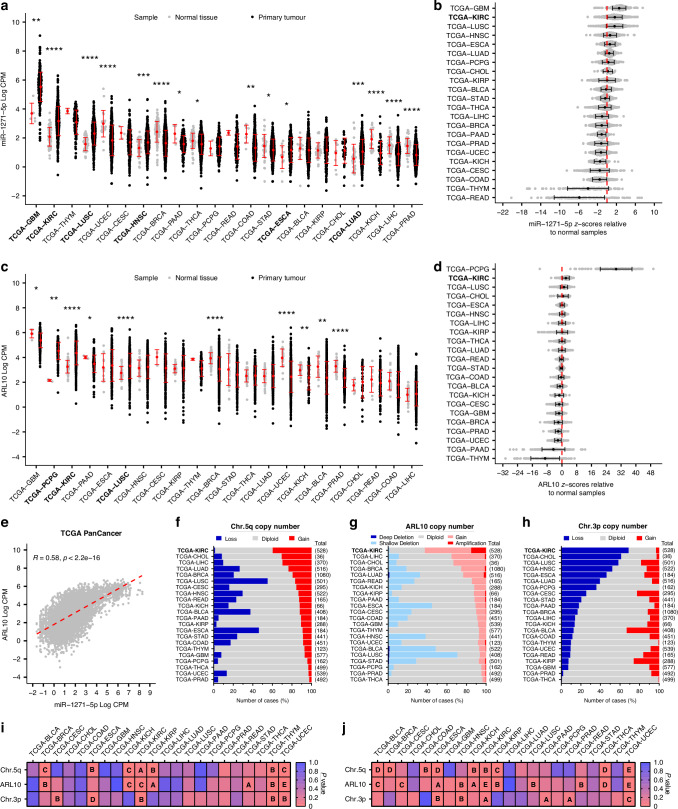
Expression of miR-1271-5p and *ARL10* among common cancer types. Expression of miR-1271-5p (**a**) and *ARL10* (**b**) across TCGA PanCancer Atlas tumours compared to their respective healthy adjacent tissues. Tumour harbouring a significant increase of miR-1271-5p or *ARL10* are marked in bold. Expression *z*-score of miR-1271-5p (**c**) and *ARL10* (**d**) in tumours compared to related normal samples. **e** Spearman correlation between the expression of miR-1271-5p and *ARL10* across all cancers. Proportion of copy number of 5q (**f**), *ARL10* (**g**) and 3p (**h**) genotype of tumours across all cancers. Correlation *p* value of miR-1271-5p (**i**) and *ARL10* (**j**) with copy number of 5q, *ARL10* and 3p. Statistical analysis was performed using Wilcoxon signed-rank tests (**a**, **b**), Spearman correlation test (**e**) or Kruskal–Wallis test (**i**, **j**). (**a** = 0.05, * or **b** < 0.05, ** or **c** < 0.01, *** or **d** < 0.001, **** or **e** < 0.0001).

Using the TCGA PanCancer Atlas, we examined whether the increases in miR-1271-5p and *ARL10* were specific to ccRCC. We only selected projects with normal adjacent tissue data. Among the 22 cancer types analysed, ccRCC tumours showed the second-highest mean expression of miR-1271-5p behind Glioblastoma Multiforme (TCGA-GBM) (Fig. 2a). Including ccRCC, seven cancer types overexpressed miR-1271-5p in their tumours compared to normal tissues. In contrast, eight types downregulated the miRNA (Fig. 2a). *ARL10* mean expression was the third highest in ccRCC tumours, following TCGA-GBM and Pheochromocytoma and Paraganglioma (TCGA-PCPG) (Fig. 2b). Only two other cancers overexpressed *ARL10*. In comparison, seven downregulated their expression (Fig. 2b). Overall, only ccRCC and Lung Squamous Cell Carcinoma (TCGA-LUSC) patients simultaneously exhibit higher levels of miR-1271-5p and *ARL10*. To better compare miR-1271-5p and *ARL10* expression changes during tumour formation across different tissues, we converted data to *z*-scores by dividing the LogCPM value of each tumour sample by the mean LogCPM of their normal tissue samples. Tumours from ccRCC patients exhibited the second-highest mean increase of both miR-1271-5p and *ARL10* expression following tumour formation (Fig. 2c, d). A Spearman correlation analysis revealed a significant positive correlation between miR-1271-5p expression and *ARL10* across all cancers, further supporting synchronous transcription (Fig. 2e).

Next, we questioned whether the genomic alterations investigated earlier are associated with miR-1271-5p and *ARL10* expression in other tumours. Among all cancers analysed, ccRCC demonstrated the highest proportion of tumours with a gain of 5q and *ARL10* and a loss of 3p (Fig. 2f–h). Most other cancers instead harboured a loss of 5q and *ARL10* (Fig. 2f, g). Furthermore, miR-1271-5p expression correlated with the copy number of either 5q, *ARL10*, or 3p in eight additional cancers (Fig. 2i). Only TCGA-KICH and TCGA-UCEC showed increased expression after the gain of 5q or ARL10 compared to diploid tumours. (Supplementary Fig. 1a, b). However, TCGA-ESCA, TCGA-KIRP, and TCGA-STAD showed decreased miR-1271-5p expression with the loss of either 5q or *ARL10* (Supplementary Fig. 1a, b). In addition, three cancers exhibited a positive correlation with the loss of 3p, including TCGA-CESC, TCGA-ESCA and TCGA-UCEC (Supplementary Fig. 2a). Regarding *ARL10*, its expression correlated with the copy number of either 5q, *ARL10*, or 3p in fourteen other cancers (Fig. 2j). Only TCGA-CESC, TCGA-READ, and TCGA-UCEC exhibited higher ARL10 expression associated with a copy number gain of ARL10 compared to diploid tumours. In contrast, TCGA-BLCA, TCGA-ESCA, TCGA-HNSC, TCGA-KICH, TCGA-KIRP, and TCGA-LUSC all showed reduced ARL10 levels following copy number loss (Supplementary Fig. 3a, b). Moreover, TCGA-ESCA, TCGA-HNSC, and TCGA-UCEC also displayed higher *ARL10* expression associated with loss of 3p compared to diploid tumours (Supplementary Fig. 2b). Altogether, the genomic events of 3p loss and copy number gain of 5q and *ARL10* are associated with the expression of miR-1271-5p and *ARL10* in various cancers. However, the higher occurrence of these genomic events in ccRCC tumours could promote an environment that simultaneously enhances miR-1271-5p and *ARL10* expression.

### The VHL/HIFα axis controls miR-1271-5p and *ARL10* expression

**Fig. 3:**
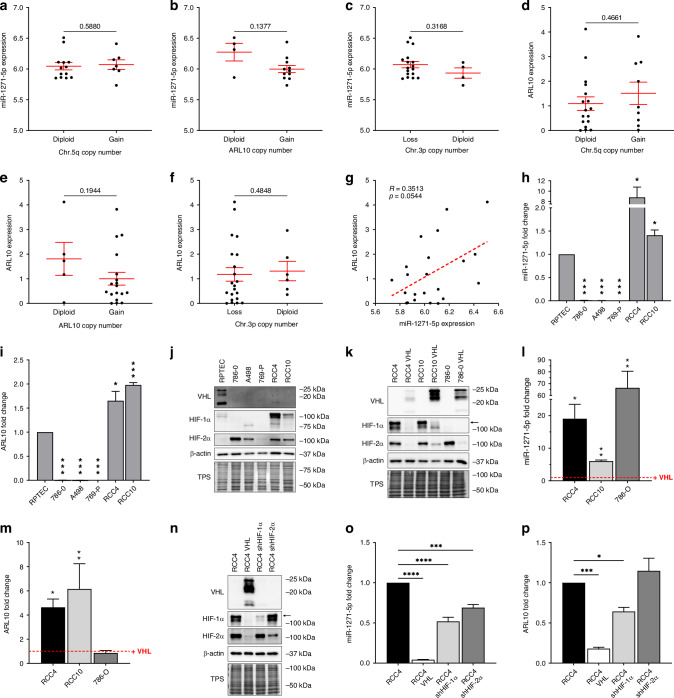
MiR-1271-5p and *ARL10* expression is controlled by the VHL/HIFα axis. Expression of miR-1271-5p in kidney cell lines based on the copy number of 5q (**a**), ARL10 (**b**) or 3p (**c**) genotype. Expression of *ARL10* in kidney cell lines based on copy number of 5q (**d**), ARL10 (**e**) or 3p (**f**) genotype. **g** Spearman correlation between the expression of miR-1271-5p and *ARL10* in kidney cell lines. RT-qPCR quantification of miR-1271-5p (**h**) and *ARL10* (**i**) along with western blot analysis (**j**) of VHL, HIF-1α (arrow) and HIF-2α in multiple kidney cell lines (RPTEC, 786-0, A498, 769-P, RCC4, RCC10) (*N* = 3–4). Western blot analysis (**k**) of VHL, HIF-1α (arrow), and HIF-2α along with RT-qPCR quantification of miR-1271-5p (**l**) and *ARL10* (**m**) in RCC4, RCC10, and 786-0 cells, with or without VHL (*N* = 3). Western blot analysis (**n**) of VHL, HIF-1α (arrow), and HIF-2α, along with RT-qPCR quantification of miR-1271-5p (**o**) and *ARL10* (**p**) in RCC4, RCC4 VHL, RCC4 shHIF-1α, and RCC4 shHIF-2α (*N* = 3). β-actin and total protein stain (TPS) were used as loading controls for the western blot experiments. MiRNA and gene expression were normalised with RNU44 and RPLPO, respectively. The data are presented as the mean ± SEM. Statistical analysis was performed using Wilcoxon signed-rank tests (**a**–**f**), Spearman correlation test (**g**), two-tailed unpaired Student’s *t*-tests (**h**, **i**, **l**, **m**) or one-way ANOVA with Dunnett’s multiple comparison test compared to RCC4 cells (**o**, **p**). (*<0.05, **<0.01, ***<0.001, ****<0.0001).

We examined the correlations between these genomic events and the expression of miR-1271-5p and *ARL10* in kidney cell lines. Using the Cancer Cell Line Encyclopedia database, we analysed all Renal Cell Carcinoma cell lines (*N* = 54). Our results indicated no difference in miR-1271-5p and *ARL10* expression between cells with a loss of 3p or a copy gain of 5q and *ARL10* (Fig. 3a–f). Although not significant, miR-1271-5p expression positively correlated with *ARL10* expression in kidney cells (Fig. 3g). To confirm that miR-1271-5p and *ARL10* levels were higher in ccRCC cell lines compared to normal cells, we measured their expression across multiple cancer models (786-0, A498, 769-P, RCC4, RCC10) relative to normal kidney cells RPTEC. Surprisingly, miR-1271-5p and *ARL10* were only upregulated in two cell lines (RCC4, RCC10) and downregulated in the other three (786-0, A498, 769-P) (Fig. 3h, i).

Since the genomic status explored earlier might not explain these discrepancies, we questioned whether the VHL/HIFα axis could be involved, as we previously identified miR-1271-5p in our VHL-dependent miRNA profiling [30]. Although all cell lines harbour VHL mutations, they exhibit distinct patterns of HIFα isoforms. RCC4 and RCC10 cells contain both HIF-1α and HIF-2α proteins, while 786-0 cells only express HIF-2α (Fig. 3j). The A498 cells express a truncated HIF-1α and wild-type (WT) HIF-2α, whereas 769-P cells do not express either isoform, although a visible band for HIF-2α can be seen (Fig. 3j) [12, 40]. We first evaluated the impact of VHL on miR-1271-5p and *ARL10* expression in RCC4, RCC10, and 786-0 cell lines alongside their WT VHL functional counterparts (RCC4 VHL, RCC10 VHL, and 786-0 VHL) (Fig. 3k). The absence of VHL significantly increased miR-1271-5p expression in all three cell lines (Fig. 3l). Similarly, *ARL10* expression was notably elevated in RCC4 and RCC10, but no changes were observed in 786-0 cells, regardless of VHL presence (Fig. 3m). Subsequently, we employed short-hairpin RNA to independently inhibit HIF-1α and HIF-2α in RCC4 cells (Fig. 3n, Supplementary Fig. 4). We found that inhibiting either HIF-1α or HIF-2α significantly decreased miR-1271-5p expression in RCC4 cells (Fig. 3o). However, only inhibiting HIF-1α, not HIF-2α, reduced *ARL10* expression (Fig. 3p). These findings may explain why *ARL10* was not affected by reintroducing VHL in 786-0 cells, which only express HIF-2α (Fig. 3h, m). Overall, these results suggest that the correlation between truncal genomic events and the expression of miR-1271-5p and *ARL10* is not directly observable in cancer cell lines. However, the loss of VHL, linked to 3p deletion, and the resulting rise in HIFα levels, are key factors in the overexpression of miR-1271-5p and ARL10 in ccRCC tumours. While HIF-1α alone seems to regulate ARL10 expression, the simultaneous presence of both HIF-1α and HIF-2α appears necessary for increased miR-1271-5p levels.

### Kidney-specific HIFα’s promoting effect on miR-1271-5p and *ARL10*

**Fig. 4:**
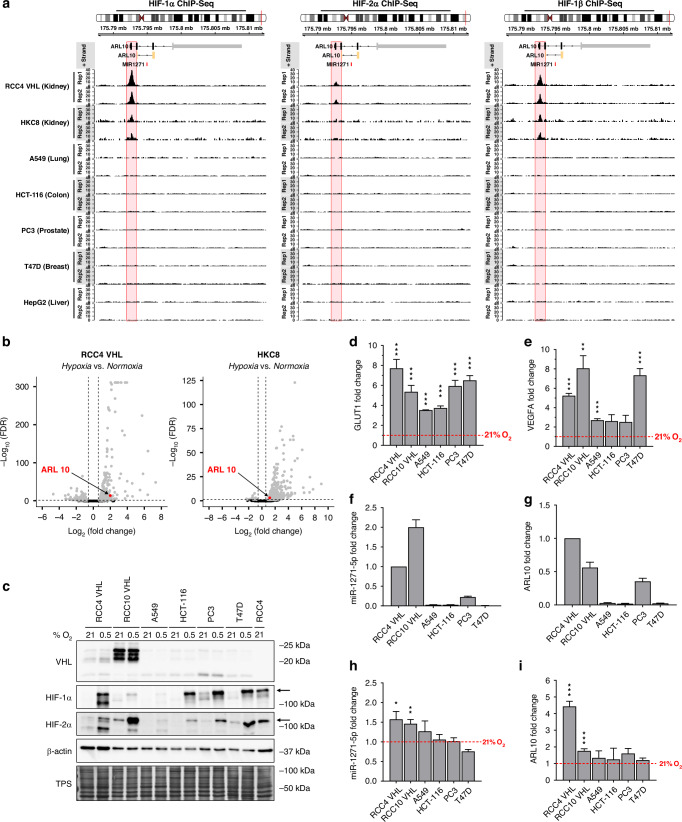
Kidney-specific HIFα promoting effect on miR-1271-5p and *ARL10.* **a** Graphical representation of HIF-1α (left), HIF-2α (middle), and HIF-1β (right) ChIP-seq analysis (GSE200205, GSE120887, GSE130989) focused on the *ARL10*/miR-1271-5p genomic location (GRCH37, Chr.5:175787500-175812500) across different cell lines. **b** Volcano plot summarising gene expression profiles following hypoxia in RCC4 VHL (left) and HKC8 (right). Genes with an absolute FC ≥ 1.5 and an FDR ≤ 0.05 were considered significant (marked in grey, whereas ARL10 is labelled in red). **c** Western blot analysis of VHL, HIF-1α (arrow), and HIF-2α (arrow) in RCC4 VHL, RCC10 VHL, A549, HCT-116, PC3, and T47D cells cultured for 16 h under normal (21% O_2_) or hypoxic (0.5% O_2_) conditions. RT-qPCR quantification of GLUT1 (**d**), VEGFA (**e**), miR-1271-5p (**f**, **h**), and ARL10 (**g**, **i**) in the cells mentioned in (**c**) (*N* = 3). β-actin and total protein stain (TPS) were used as loading controls for the western blot experiment. MiRNA and gene expression were normalised with RNU48 and RPLPO, respectively. The data are presented as Mean ± SEM. Statistical analysis was performed using two-tailed unpaired Student’s *t*-tests compared to normal conditions (21% O_2_). (*<0.05, **<0.01, ***<0.001).

To better understand how HIF influences miR-1271-5p and *ARL10* expression, we analysed PanCancer HIF ChIP-seq data (GSE120887, GSE200205, GSE130989) [34–36]. In these studies, HIF-1α, HIF-2α, and HIF-1β were independently immunoprecipitated in seven cell lines exposed to hypoxia, enabling us to determine if HIF can bind near the transcription start site (TSS) of *ARL10*/miR-1271-5p as it does with HIF-regulated genes such as Lactate Dehydrogenase A (*LDHA*) (Supplementary Fig. 5). Beginning with kidney cell lines (RCC4 VHL, HKC8), both HIF-1α and HIF-1β bind near the *ARL10* genomic location, especially in RCC4 VHL cells (Fig. 4a). In contrast, a binding signal for HIF-2α is only observable in RCC4 VHL cells. No binding signal was detected for all three HIF isoforms across other cancer cell lines (A549, HCT-116, PC3, T47D, HepG2) (Fig. 4a). RNAseq analysis confirmed that *ARL10* was significantly higher in RCC4 VHL and HKC8 cells under hypoxic conditions (Fig. 4b). Meanwhile, *ARL10* expression remained low in all other cell lines, even under hypoxia, suggesting that HIF stimulates miR-1271-5p and *ARL10* only in kidney cells.

To confirm these findings, we exposed RCC4 VHL, RCC10 VHL, A549, HCT-116, PC3, and T47D cells to hypoxia and quantified miR-1271-5p and *ARL10* expression. After 16 h at 0.5% O_2_, all six cell lines showed an increased abundance of HIF-1α and HIF-2α (Fig. 4c). Additionally, a significant increase in Solute Carrier Family 2 Member 1 (*SLC2A1*/*GLUT1*) and Vascular Endothelial Growth Factor A (*VEGFA*) genes was observed, supporting the enhanced activity of HIF (Fig. 4d, e). Moreover, we found that the basal expression of miR-1271-5p and *ARL10* was higher in ccRCC cell lines (RCC4 VHL, RCC10 VHL) compared to other cancer cell types (A549, HCT-116, PC3, T47D) at 21% O_2_, supporting previous TCGA PanCancer results (Figs. 2a and 4f, g). During hypoxia, only RCC4 VHL and RCC10 VHL cells showed significant increases in miR-1271-5p and *ARL10* (Fig. 4h, i). However, the elevation of miR-1271-5p was less pronounced than that of *ARL10*. These findings support the idea that HIF regulates both miR-1271-5p and *ARL10* and suggest that this promoting effect is specific to kidney cells.

### Identification of HRE sites near *ARL10*/miR-1271-5p

**Fig. 5:**
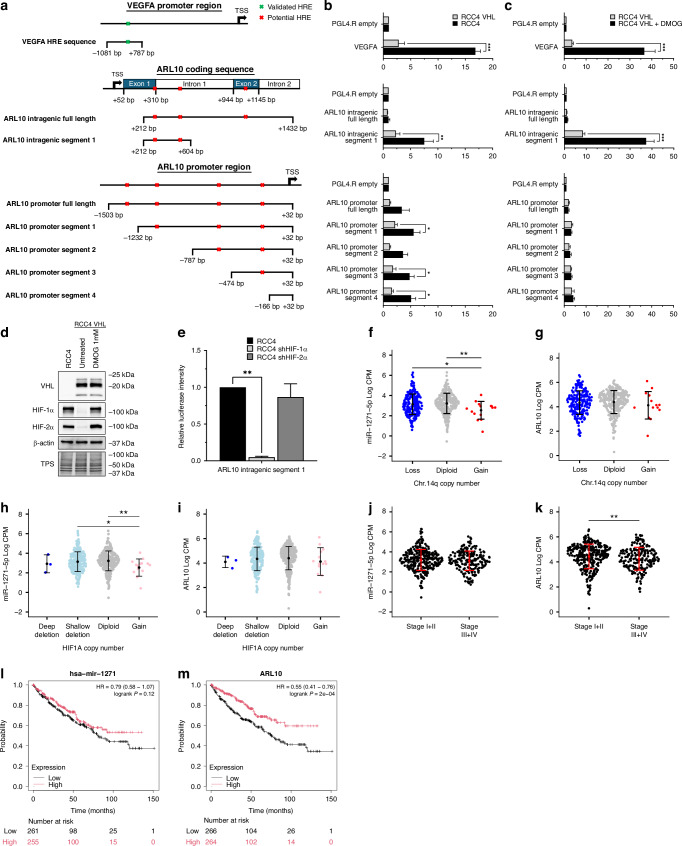
Identification of HRE sites near *ARL10*/miR-1271-5p. **a** Graphical representation of the genomic regions cloned into the Firefly luciferase reporter vector. Regions of interest include the validated VEGFA HRE sequence (top), ARL10 intragenic region (middle), and ARL10 promoter region (bottom). The validated HRE site is marked in green, while potential HRE sites are highlighted in red. **b** Relative firefly luciferase signal in RCC4 and RCC4 VHL cells compared to the empty vector. **c** Relative firefly luciferase signal in RCC4 VHL following treatment with 1 mM of DMOG (24 h) compared to the empty vector. **d** Western blot analysis of VHL, HIF-1α, and HIF-2α in RCC4 VHL, following treatment with DMOG. **e** Relative firefly luciferase signal of ARL10-Intragenic-Segment-1 in RCC4, RCC4 shHIF-1α, and RCC4 shHIF-2α. Expression of miR-1271-5p (**f**) and *ARL10* (**g**) in ccRCC tumours based on 14q copy number genotype. Expression of miR-1271-5p (**h**) and *ARL10* (**i**) in ccRCC tumours based on *HIF1A* copy number genotype. Expression of miR-1271-5p (**j**) and *ARL10* (**k**) in ccRCC tumours based on tumour stage. Survival plot of ccRCC patients (TCGA-KIRC) based on miR-1271-5p (**l**) and *ARL10* (**m**) expression. β-actin and total protein stain (TPS) were used as loading controls for the western blot experiment. The data are presented as the Mean ± SEM. Statistical analysis was performed using two-tailed unpaired Student’s *t*-tests between RCC4 and RCC4 VHL (**b**) or RCC4 VHL-treated and untreated cells (**c**), one-way ANOVA with Dunnett’s multiple comparison test compared to RCC4 cells (**e**), Wilcoxon signed-rank tests (**f**–**k**) or Kaplan–Meier survival analysis (**l**, **m**). (*<0.05, **<0.01, ***<0.001).

To confirm the direct influence of HIF, we searched for HRE sequences (5′-RCGTG-3′) near *ARL10*/miR-1271-5p. ChIP-seq results showed a peak binding signal in the first intron of *ARL10* (Fig. 4a). In fact, genomic sequence revealed three potential intragenic HRE sequences: at the end of the first exon, within the first intron, and in the second exon (Fig. 5a). Four additional HRE sites were identified in the promoter region (Fig. 5a). To confirm HIFα binding, we cloned each sequence into a Firefly luciferase-expressing plasmid with minimal promoter activity. Additionally, we dissected the promoter and intragenic regions of interest to distinguish the effect of each potential HRE site (Fig. 5a). The *VEGFA* HRE sequence served as a positive control (Fig. 5a). We used RCC4 and RCC4 VHL cells to test whether VHL loss affects the promoting ability of each region of interest. As expected, after transfecting cells with the plasmid containing the HRE of *VEGFA*, we observed higher levels of luciferase in RCC4 cells (Fig. 5b). No difference was measured in the intragenic region between cells for the full-length plasmid (ARL10 Intragenic Full Length) (Fig. 5b). However, the fragmented region (ARL10 Intragenic Segment 1) in the vector significantly increased luciferase in RCC4 cells compared to RCC4 VHL (Fig. 5b). Most tested fragments in the promoter region also showed a luciferase increase in RCC4 cells compared to RCC4 VHL (Fig. 5b). These results suggest that these genomic regions contain response elements that are activated in the absence of VHL.

To confirm that *ARL10* genomic regions contain functional HRE sequences, RCC4 VHL cells were treated with 1 mM DMOG for 24 h. Luciferase activity increased in our VEGFA control after DMOG treatment (Fig. 5c, d). In our regions of interest, DMOG treatment significantly increased luciferase concentration for the ARL10 Intragenic Segment 1 (Fig. 5c). No changes were observed in any of the tested promoter segments, suggesting there is no active HRE in the upstream promoter region of *ARL10* (Fig. 5c). We further confirmed the binding of HIF at the intragenic region by transfecting our plasmid into RCC4 shHIF-1α and RCC4 shHIF-2α cells. Consistent with previous findings, only the absence of HIF-1α markedly reduced luciferase expression, confirming the presence of an HIF-1α HRE sequence within the intragenic region of *ARL10* (Fig. 5e). As mentioned previously, loss of chromosome 14q, which contains *HIF1A*, occurs frequently in ccRCC tumours and correlates with tumour stage. Therefore, we examined whether miR-1271-5p and *ARL10* expression also correlate with 14q loss or focal HIF1A copy number loss, as well as tumour stage. Initially, neither miR-1271-5p nor *ARL10* expression levels showed any association with 14q copy number loss or *HIF1A* (Fig. 5f–i). However, *ARL10*, unlike miR-1271-5p, was significantly decreased in more advanced tumours (Fig. 5j, k). Consistently, higher *ARL10* levels positively correlated with improved overall survival in ccRCC patients (Fig. 5l), whereas no such relationship was observed for miR-1271-5p (Fig. 5m). Collectively, these results confirm that the overexpression of miR-1271-5p and *ARL10* in ccRCC is driven by HIF-1α binding near the junction between the first exon and intron of *ARL10*. While miR-1271-5p expression remains stable throughout the disease, *ARL10* expression emerges as a potential prognostic marker in ccRCC.

## Discussion

Multiregional deep-sequencing studies have advanced the understanding of ccRCC evolution. Loss of chromosome 3p is the initial step towards tumour formation and can occur years before cancer diagnosis [21]. Indeed, key driver mutations that later guide clonal expansion primarily affect crucial tumour suppressor genes located on 3p, such as *VHL*, *PBRM1*, *SETD2*, and *BAP1* [7, 8]. Loss of 3p heterogeneity is mainly due to an unbalanced translocation involving the terminal end of 5q [21]. Copy number gain of proto-oncogenes on 5q could contribute to ccRCC tumorigenesis; for example, *SQSTM1*/p62 is overexpressed following 5q gain in ccRCC, and its loss disrupts tumour growth by regulating key oncogenic pathways such as NRF2, NF-kB, and mTOR [22]. Interestingly, these pathways regulate autophagy, a process that has therapeutic potential in VHL-deficient ccRCC [41]. Additionally, *VCAN* correlates with 5q gain and promotes ccRCC cell migration and invasion [23, 24]. To complement these findings, we investigated whether miRNAs on chromosome 5q were affected in ccRCC tumours. Differential expression analysis from TCGA revealed that five miRNAs located at the end of chromosome 5q were elevated in tumours. However, only one miRNA, miR-1271-5p, showed a positive correlation with both 5q gain and 3p loss. Moreover, the host gene, *ARL10*, was also significantly increased in tumours and correlated with these genomic alterations. A previous profiling from our lab showed miR-1271-5p overexpression in the absence of *VHL* [30]. Here, we confirmed that both miR-1271-5p and *ARL10* increased due to HIF-1α stabilisation and direct binding via internal HRE sites. These results demonstrate that 5q gain and *VHL* loss, two key truncal events, can promote the expression of miR-1271-5p and *ARL10*, uncovering a potential diagnostic tool for ccRCC tumours.

In most cancers, miR-1271-5p is widely regarded as a tumour-suppressive miRNA. Our PanCancer analysis supported the downregulation of the miRNA in several cancers, including Endometrial Cancer [42, 43], Breast Cancer [44, 45], and Prostate Cancer [46, 47]. Although we observed a notable increase in Lung Cancer and Glioblastoma, miR-1271-5p has mainly been shown to function as a tumour suppressor [48–51]. In ccRCC, two out of three groups previously indicated an increase of miR-1271-5p levels in tumours compared to normal adjacent tissues [52–54]. Results from our patient cohort, as well as those from the TCGA-KIRC, are consistent with the increase of miR-1271-5p in ccRCC tumours. Although Zhou et al. previously demonstrated that miR-1271-5p was downregulated in ccRCC cell lines, their studies were conducted in cells that either lacked HIF-1α (786-0) or expressed a functional *VHL* gene (Caki-1, ACHN) [55, 56]. As demonstrated in this study, miR-1271-5p expression was significantly increased only in RCC4 and RCC10 cells lacking VHL and co-expressing both HIF-1α and HIF-2α, compared to renal proximal tubule epithelial cells. Silencing either HIF-1α or HIF-2α led to a significant reduction in miR-1271-5p levels. These findings underscore the critical role of VHL loss and the resulting accumulation of both HIFα isoforms in driving miR-1271-5p upregulation in ccRCC cells.

Only one previous cancer-related study conducted in cutaneous melanoma indicated that *ARL10* was downregulated in primary and metastatic tumour tissue [57]. Our analysis revealed an additional increase in *ARL10* in Pheochromocytoma and Paraganglioma (PPGL) and in lung carcinoma. Interestingly, PPGL tumours often harbour alterations in the VHL/HIF pathway, which could explain the elevated expression of *ARL10* [58]. Aside from cancer, the biological function of *ARL10* remains poorly understood. The gene encodes an ARF family member, a small GTPase. Using a proximity-dependent biotin identification approach, Quirion and colleagues indicated that ARL10 could accumulate in the mitochondria, peroxisome, endoplasmic reticulum, and nucleus [59]. Furthermore, ARL10 could interact with multiple proteins, including members of the multiprotein mTORC2 complex. However, it remains unknown whether ARL10 interaction influences the functional role of mTORC2. Previous studies have shown that miR-1271-5p suppresses tumours by inhibiting the mTOR pathway, either by directly targeting mTOR [46, 60–62] or by hindering its activators such as PDK1 [63]. Activating mutations of mTOR are common in patients with ccRCC, and agents that directly target mTOR, like Everolimus, are used for treatment [4]. Others have demonstrated that 5q gain intensifies mTOR activity by increasing oncogenes such as SQSTM1 and GNB2L1/RACK1 [22, 64]. Further investigation would be interesting to better understand how the increase of miR-1271-5p and *ARL10* relates to mTOR function in ccRCC tumours.

Although the mechanistic role of miR-1271-5p and *ARL10* in ccRCC remains unclear, the kidney-specific influence of HIF-1α on their expression is intriguing. In ccRCC tumours, HIF-1α is more associated with tumour suppression, while HIF-2α appears to have a prominent oncogenic role [65, 66]. Loss of chromosome 14q, which contains the *HIF1A* gene, has been associated with a more aggressive disease [12, 67, 68]. While HIF-1α plays a key role in regulating the expression of both miR-1271-5p and *ARL10*, the loss of 14q was not linked to decreased levels of either. However, *ARL10* levels decreased in higher-grade tumours and were positively correlated with better survival rates. In contrast, miR-1271-5p expression remained relatively stable across advanced tumour stages. Compared with *ARL10*, HIF-2α also contributed to miR-1271-5p expression. The influence of HIF-2α is likely indirect, as no binding was observed at the identified internal HRE sites. Still, HIF-2α may contribute to the consistent increase of miR-1271-5p, even as HIF-1α activity declines as the disease progresses.

Previous work by Cochetti and colleagues identified a panel of three miRNAs, miR-1271, miR-122 and miR-15b, as potential urinary biomarkers for early ccRCC detection [69, 70]. Our research demonstrated that miR-1271-5p is also elevated in plasma samples from ccRCC patients, further underscoring its diagnostic potential. As demonstrated by Cochetti and other groups, using multiple miRNAs simultaneously offers greater accuracy and improved prognostic capabilities than measuring a single miRNA [69–72]. Here, we provided evidence that miR-1271-5p and *ARL10* increase in only a small subset of cancers and correlate with key genetic alterations that are enriched in ccRCC tumours. Furthermore, the kidney-specific influence of HIF-1α towards *ARL10* expression could serve as a useful prognostic marker as it positively correlates with overall survival. Finally, miR-1271-5p and *ARL10* could also help distinguish between different ccRCC subtypes since the increase was only observed in ccRCC tumours. Overall, liquid biopsies using plasma and urine are becoming increasingly attractive as easy, non-invasive diagnostic tools in cancer research, and we suggest including miR-1271-5p in future panels for ccRCC patients.

## Conclusions

In this study, we further explored the oncogenic influence of chromosome 5q gain in ccRCC tumours by investigating the expression of local miRNAs. We discovered that overexpression of miR-1271-5p and its host gene, *ARL10*, was directly associated with classic ccRCC truncal events, such as 3p loss, 5q gain, and VHL loss. Furthermore, the direct promoting effect of HIF-1α on miR-1271-5p expression after VHL loss was specific to kidney cells. These observations suggest that the evolutionary trajectory of ccRCC tumours leads to a distinctive miR-1271-5p signature compared to other tumours. This statement, combined with higher levels of miR-1271-5p in plasma samples, further supports its potential as an early biomarker for ccRCC patients.

## Supplementary information

Supplementary Tables

Supplementary Figure 1

Supplementary Figure 2

Supplementary Figure 3

Supplementary Figure 4

Supplementary Figure 5

## Data Availability

This published article and its supplementary information files include all data generated or analysed during this study.
