# Supplementary material for: Kidney-specific HIF-1α-dependent ARL10/miR-1271-5p overexpression in clear cell renal cell carcinoma
Source: Br J Cancer. 2026 Apr 17;135(2):189–201. doi: 10.1038/s41416-026-03399-w (PMC13310816; doi:10.1038/s41416-026-03399-w)
Supplement: Supplementary file 1 — Supplementary Tables [file 41416_2026_3399_MOESM1_ESM.docx]

**Supplementary tables**

Supplementary Table 1: Copy number of chromosome 5q across the TCGA PanCancer Atlas

| **Cancer type** | **Copy number of chromosome 5q** | | | |
| --- | --- | --- | --- | --- |
|  | **Loss** | **Diploid** | **Gain** | **Total** |
| TCGA-BLCA | 153 | 206 | 49 | 408 |
| TCGA-BRCA | 221 | 649 | 210 | 1080 |
| TCGA-CESC | 47 | 202 | 46 | 295 |
| TCGA-CHOL | 3 | 22 | 11 | 36 |
| TCGA-COAD | 78 | 334 | 39 | 451 |
| TCGA-ESCA | 85 | 82 | 17 | 184 |
| TCGA-GBM | 45 | 491 | 41 | 577 |
| TCGA-HNSC | 154 | 298 | 70 | 522 |
| TCGA-KICH | 10 | 48 | 8 | 66 |
| TCGA-KIRC | 7 | 311 | 210 | 528 |
| TCGA-KIRP | 8 | 249 | 31 | 288 |
| TCGA-LIHC | 36 | 224 | 110 | 370 |
| TCGA-LUAD | 137 | 257 | 122 | 516 |
| TCGA-LUSC | 276 | 141 | 84 | 501 |
| TCGA-PAAD | 9 | 155 | 20 | 184 |
| TCGA-PCPG | 7 | 148 | 7 | 162 |
| TCGA-PRAD | 22 | 458 | 12 | 492 |
| TCGA-READ | 38 | 106 | 21 | 165 |
| TCGA-STAD | 106 | 296 | 39 | 441 |
| TCGA-THCA | 0 | 482 | 17 | 499 |
| TCGA-THYM | 1 | 113 | 9 | 123 |
| TCGA-UCEC | 72 | 450 | 17 | 539 |

Supplementary Table 2: Copy number of *ARL10* across the TCGA PanCancer Atlas

| **Cancer type** | **Copy number of *ARL10*** | | | | | |
| --- | --- | --- | --- | --- | --- | --- |
|  | **Deep Deletion** | **Shallow Deletion** | **Diploid** | **Gain** | **Amplification** | **Total** |
| TCGA-BLCA | 5 | 193 | 181 | 27 | 2 | 408 |
| TCGA-BRCA | 2 | 200 | 602 | 261 | 15 | 1080 |
| TCGA-CESC | 0 | 70 | 195 | 29 | 1 | 295 |
| TCGA-CHOL | 0 | 3 | 21 | 12 | 0 | 36 |
| TCGA-COAD | 0 | 89 | 321 | 40 | 1 | 451 |
| TCGA-ESCA | 0 | 82 | 82 | 19 | 1 | 184 |
| TCGA-GBM | 1 | 55 | 474 | 47 | 0 | 577 |
| TCGA-HNSC | 0 | 199 | 285 | 38 | 0 | 522 |
| TCGA-KICH | 0 | 9 | 49 | 8 | 0 | 66 |
| TCGA-KIRC | 0 | 7 | 190 | 250 | 81 | 528 |
| TCGA-KIRP | 0 | 11 | 241 | 33 | 3 | 288 |
| TCGA-LIHC | 0 | 40 | 199 | 126 | 5 | 370 |
| TCGA-LUAD | 6 | 161 | 228 | 108 | 13 | 516 |
| TCGA-LUSC | 5 | 349 | 115 | 32 | 0 | 501 |
| TCGA-PAAD | 1 | 19 | 142 | 20 | 2 | 184 |
| TCGA-PCPG | 0 | 9 | 145 | 7 | 1 | 162 |
| TCGA-PRAD | 0 | 16 | 452 | 21 | 3 | 492 |
| TCGA-READ | 0 | 44 | 97 | 24 | 0 | 165 |
| TCGA-STAD | 3 | 123 | 282 | 28 | 5 | 441 |
| TCGA-THCA | 0 | 0 | 480 | 17 | 2 | 499 |
| TCGA-THYM | 0 | 1 | 113 | 9 | 0 | 123 |
| TCGA-UCEC | 0 | 73 | 421 | 39 | 6 | 539 |

Supplementary Table 3: Copy number of chromosome 3p across the TCGA PanCancer Atlas

| **Cancer type** | **Copy number of chromosome 3p** | | | |
| --- | --- | --- | --- | --- |
|  | **Loss** | **Diploid** | **Gain** | **Total** |
| TCGA-BLCA | 51 | 221 | 136 | 408 |
| TCGA-BRCA | 179 | 775 | 126 | 1080 |
| TCGA-CESC | 75 | 153 | 67 | 295 |
| TCGA-CHOL | 22 | 13 | 1 | 36 |
| TCGA-COAD | 51 | 356 | 44 | 451 |
| TCGA-ESCA | 85 | 71 | 28 | 184 |
| TCGA-GBM | 41 | 478 | 58 | 577 |
| TCGA-HNSC | 272 | 183 | 67 | 522 |
| TCGA-KICH | 9 | 49 | 8 | 66 |
| TCGA-KIRC | 363 | 148 | 17 | 528 |
| TCGA-KIRP | 21 | 192 | 75 | 288 |
| TCGA-LIHC | 52 | 283 | 35 | 370 |
| TCGA-LUAD | 203 | 275 | 38 | 516 |
| TCGA-LUSC | 292 | 116 | 93 | 501 |
| TCGA-PAAD | 33 | 142 | 9 | 184 |
| TCGA-PCPG | 58 | 104 | 0 | 162 |
| TCGA-PRAD | 9 | 443 | 40 | 492 |
| TCGA-READ | 15 | 123 | 27 | 165 |
| TCGA-STAD | 90 | 306 | 45 | 441 |
| TCGA-THCA | 3 | 493 | 3 | 499 |
| TCGA-THYM | 12 | 110 | 1 | 123 |
| TCGA-UCEC | 51 | 440 | 48 | 539 |

Supplementary Table 4: List of primers used for qPCR assays

| **Genes** | **Forward primer (5’-3’)** | **Reverse primer (5’-3’)** |
| --- | --- | --- |
| ARL10 | TACTGGAAGGAGTTTGTGAGCG | TCCCCCATACTCATGGCCTC |
| GLUT1 | TCTGGCATCAACGCTGTCTTC | CGATACCGGAGCCAATGGT |
| VEGFA | AGGGCAGAATCATCACGAAGT | AGGGTCTCGATTGGATGGCA |
| BNIP3 | TGAGTCTGGACGGAGTAGCTC | CCCTGTTGGTATCTTGTGGTGT |
| TGFα | AGGTCCGAAAACACTGTGAGT | AGCAAGCGGTTCTTCCCTTC |
| RPLPO | GCAGCATCTACAACCCTGAAG | CACTGGCAACATTGCGGAC |
| HPRT1 | GAAAAGGACCCCACGAAGTGT | AGTCAAGGGCATATCCTACAACA |
| **miRNAs** | **Catalog number (Thermo Fisher Scientific)** | |
| miR-1271-5p | 002779 | |
| RNU44 | 001094 | |
| RNU48 | 001006 | |
| Cel-miR-39-3p | 000200 | |

Supplementary Table 5: Culture media and supplements used for each cell lines

| **Cell line** | **Media** | **Supplements** |
| --- | --- | --- |
| RCC4  RCC10  786-0  A549 | DMEM/high glucose  (#319-030-CL)^1^ | - 10% Fetal Bovine Serum^1^ - 2mM L-glutamine^1^ - 1mM Sodium Pyruvate^2^ |
| A498 | EMEM  (#320-005-CL)^1^ | - 10% Fetal Bovine Serum^1^ - 1mM Sodium Pyruvate^2^ - 1X MEM Non-Essential Amino Acids^3^ |
| 769-P | RPMI 1640  (#350-007-CL)^1^ | - 10% Fetal Bovine Serum^1^ - 1mM Sodium Pyruvate^2^ |
| HCT-116 | McCoy’s 5A  (#317-010-CL)^1^ | - 10% Fetal Bovine Serum^1^ |
| PC3 | RPMI 1640  (#350-007-CL)^1^ | - 10% Fetal Bovine Serum^1^ |
| T47D | RPMI 1640  (#350-007-CL)^1^ | - 10% Fetal Bovine Serum^1^ - 10 µg/mL Insulin^4^ |
| RPTEC | Renal Epithelial Cell Growth Medium 2  (C-26030)^5^ | - Premixed supplements^5^ |

^1^Wisent Bioproducts

^2^Cytiva

^3^Thermo Scientific

^4^ Sigma-Aldrich

^5^ PromoCell

Supplementary Table 6: List of primers used for cloning HRE containing regions

| **Region of interest** | **Forward primer (5’-3’)** | **Reverse primer (5’-3’)** |
| --- | --- | --- |
| VEGFA HRE sequence | TGGCCTAACTGGCCGGTACACGCGTTGAATCATCACGCAGGCC | TATATACCCTCTAGTGTCTAAGCTTGGGTCCTTTGGGAAGTGTC |
| ARL10 Promoter Full Length | TGGCCTAACTGGCCGGTACACGCGTATTCTCCTGCCTCAGTCTACC | TATATACCCTCTAGTGTCTAAGCTTACCCACCCAGACCCAGTC |
| ARL10 Promoter Segment 1 | TGGCCTAACTGGCCGGTACACGCGTACGGATTCTTGCTCTGTCACC | TATATACCCTCTAGTGTCTAAGCTTACCCACCCAGACCCAGTC |
| ARL10 Promoter Segment 2 | TGGCCTAACTGGCCGGTACACGCGTGCCAGGGGTACAGAGGAATC | TATATACCCTCTAGTGTCTAAGCTTACCCACCCAGACCCAGTC |
| ARL10 Promoter Segment 3 | TGGCCTAACTGGCCGGTACACGCGTGACCCCAGCAAAACCCCTG | TATATACCCTCTAGTGTCTAAGCTTACCCACCCAGACCCAGTC |
| ARL10 Promoter Segment 4 | TGGCCTAACTGGCCGGTACACGCGTGTGGATGCTAGCAAGCACC | TATATACCCTCTAGTGTCTAAGCTTACCCACCCAGACCCAGTC |
| ARL10 Intragenic Full Length | TGGCCTAACTGGCCGGTACACGCGTAAGACCTACTTCGGCCGC | TATATACCCTCTAGTGTCTAAGCTTAGTGCTTGGCATATGAACCC |
| ARL10 Intragenic Segment 1 | TGGCCTAACTGGCCGGTACACGCGTAAGACCTACTTCGGCCGC | TATATACCCTCTAGTGTCTAAGCTTCTCCCGAGCCGCGCCCTC |
| RVprimer3 | CTAGCAAAATAGGCTGTCCC | --- |

Supplementary table 7: Clinicopathological characteristics of ccRCC patients

| **Characteristics** | **CHX**  (N =20) | **MET**  (N=20) |
| --- | --- | --- |
| **Samples** |  |  |
| Plasma | 20 | 20 |
| Primary Tumor | 20 | 0 |
| Normal Adjacent Tissue | 20 | 0 |
| **Gender** |  |  |
| Men | 11 | 17 |
| Women | 7 | 3 |
| Unknown | 2 | 0 |
| **Age** |  |  |
| ≥ 64 | 9 | 15 |
| < 64 | 9 | 5 |
| Unknown | 2 | 0 |
| **Tumor Size Radiology** |  |  |
| Average (cm^3^) | 8.04 | 8.00 |
| Median (cm^3^) | 6.30 | 7.10 |
| Unknown | 2 | 0 |
| **Tumor Size Pathology** |  |  |
| Average (cm^3^) | 7.26 | 6.77 |
| Median (cm^3^) | 6.15 | 6.00 |
| Unknown | 2 | 7 |
| **Tumor Grade** |  |  |
| T1 | 4 | 3 |
| T2 | 0 | 0 |
| T3 | 13 | 9 |
| T4 | 1 | 1 |
| Unknown | 2 | 7 |
| **Lymph Node Metastasis** |  |  |
| N0 | 5 | 1 |
| NX | 13 | 12 |
| Unknown | 2 | 7 |
| **Distant Metastasis** |  |  |
| Yes | 5 | 20 |
| No | 13 | 0 |
| Unknown | 2 | 0 |

**Supplementary Figure legends**

Supplementary Figure 1: PanCancer miR-1271-5p expression profiles based on chromosome 5q and *ARL10* copy number

**A-B)** Expression of miR-1271-5p in tumor samples based on their copy number of 5q (A) and *ARL10* (B) genotype across multiple common cancers. Statistical analysis was performed using Wilcoxon signed-rank tests. (* < 0.05, ** < 0.01, *** < 0.001).

Supplementary Figure 2: PanCancer miR-1271-5p and *ARL10* expression profiles based on chromosome 3p copy number

**A-B)** Expression of miR-1271-5p (A) and *ARL10* (B) in tumor samples based on their copy number of 3p genotype across multiple common cancers. Statistical analysis was performed using Wilcoxon signed-rank tests. (* < 0.05, ** < 0.01, *** < 0.001).

Supplementary Figure 3: PanCancer *ARL10* expression profiles based on chromosome 5q and *ARL10* copy number

**A-B)** Expression *ARL10* in tumor samples based on their copy number of 5q (A) and *ARL10* (B) genotype across multiple common cancers. Statistical analysis was performed using Wilcoxon signed-rank tests. (* < 0.05, ** < 0.01, *** < 0.001, **** < 0.0001).

Supplementary Figure 4: HIF-related target quantification

**A-B)** RT-qPCR quantification of *BNIP3* (A) and *TGFα* (B) in RCC4, RCC4 VHL, RCC4 shHIF-1α, and RCC4 shHIF-2α (N=3). Statistical analysis was performed using one-way ANOVA with Dunnett’s multiple comparison test compared to RCC4 cells. (* < 0.05, ** < 0.01, *** < 0.001, **** < 0.0001).

Supplementary Figure 5: HIF ChIP-seq near LDHA

Graphical representation of HIF-1α (left), HIF-2α (middle), and HIF-1β (right) ChIP-seq analysis (GSE200205, GSE120887, GSE130989) focused on the *LDHA* genomic location (GRCH37, Chr.11: 18410000- 18431000) across different cell lines.
