# Supplementary figures and images for: Kidney-specific HIF-1α-dependent ARL10/miR-1271-5p overexpression in clear cell renal cell carcinoma
Source: Br J Cancer. 2026 Apr 17;135(2):189–201. doi: 10.1038/s41416-026-03399-w (PMC13310816; doi:10.1038/s41416-026-03399-w)

**A)**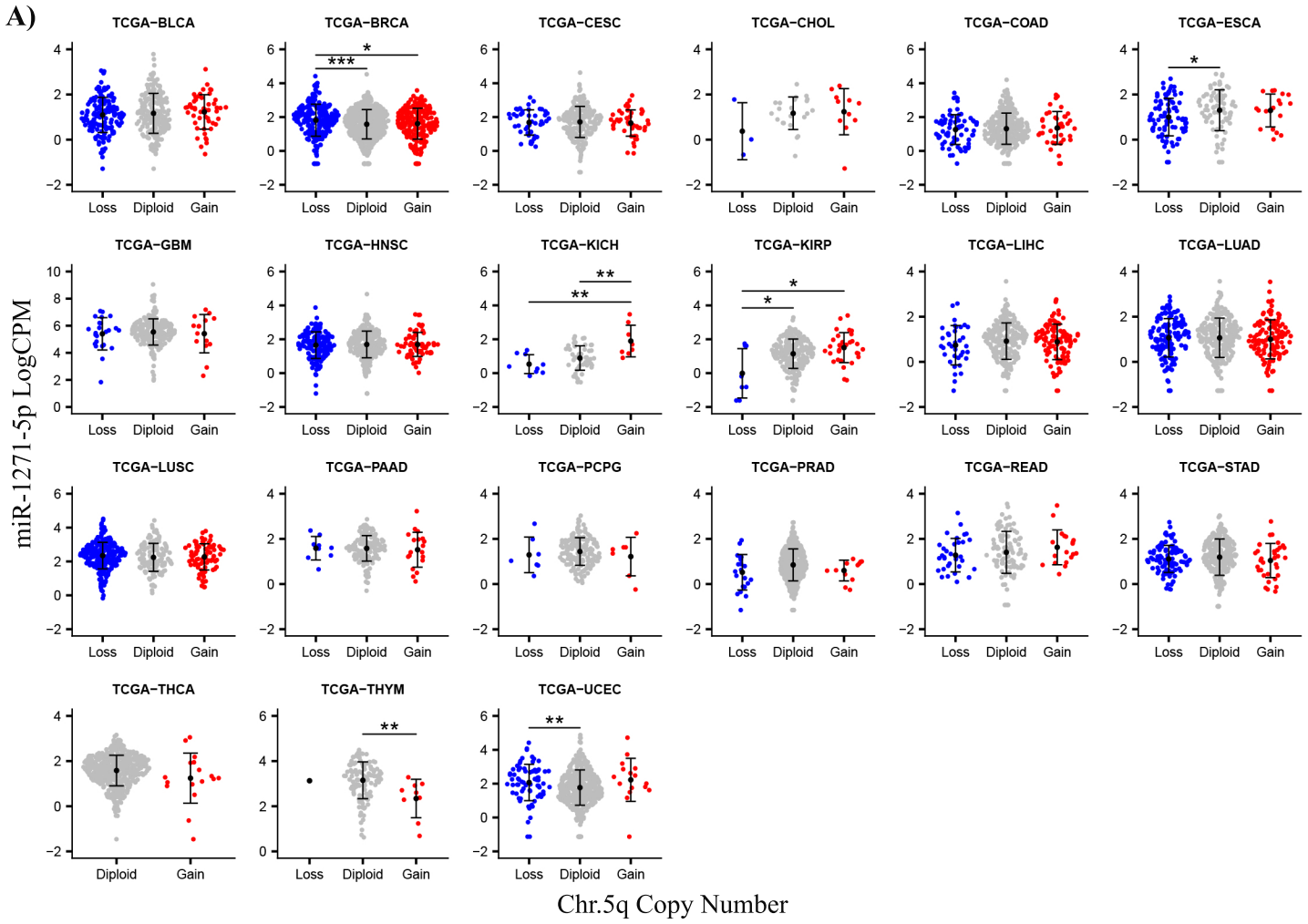**B)**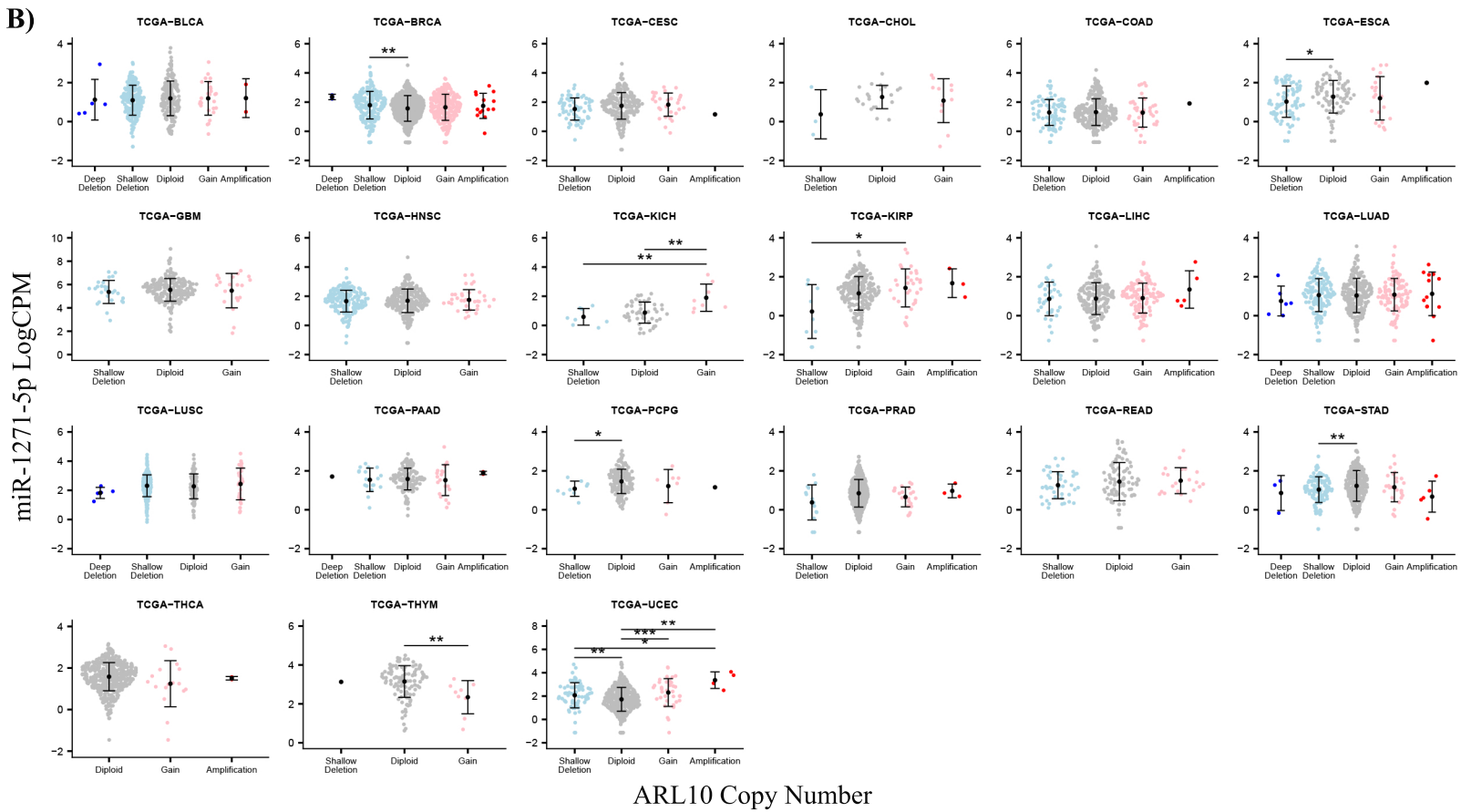

Supplement: Supplementary file 2 — Supplementary Figure 1 [file 41416_2026_3399_MOESM2_ESM.pdf]

A)

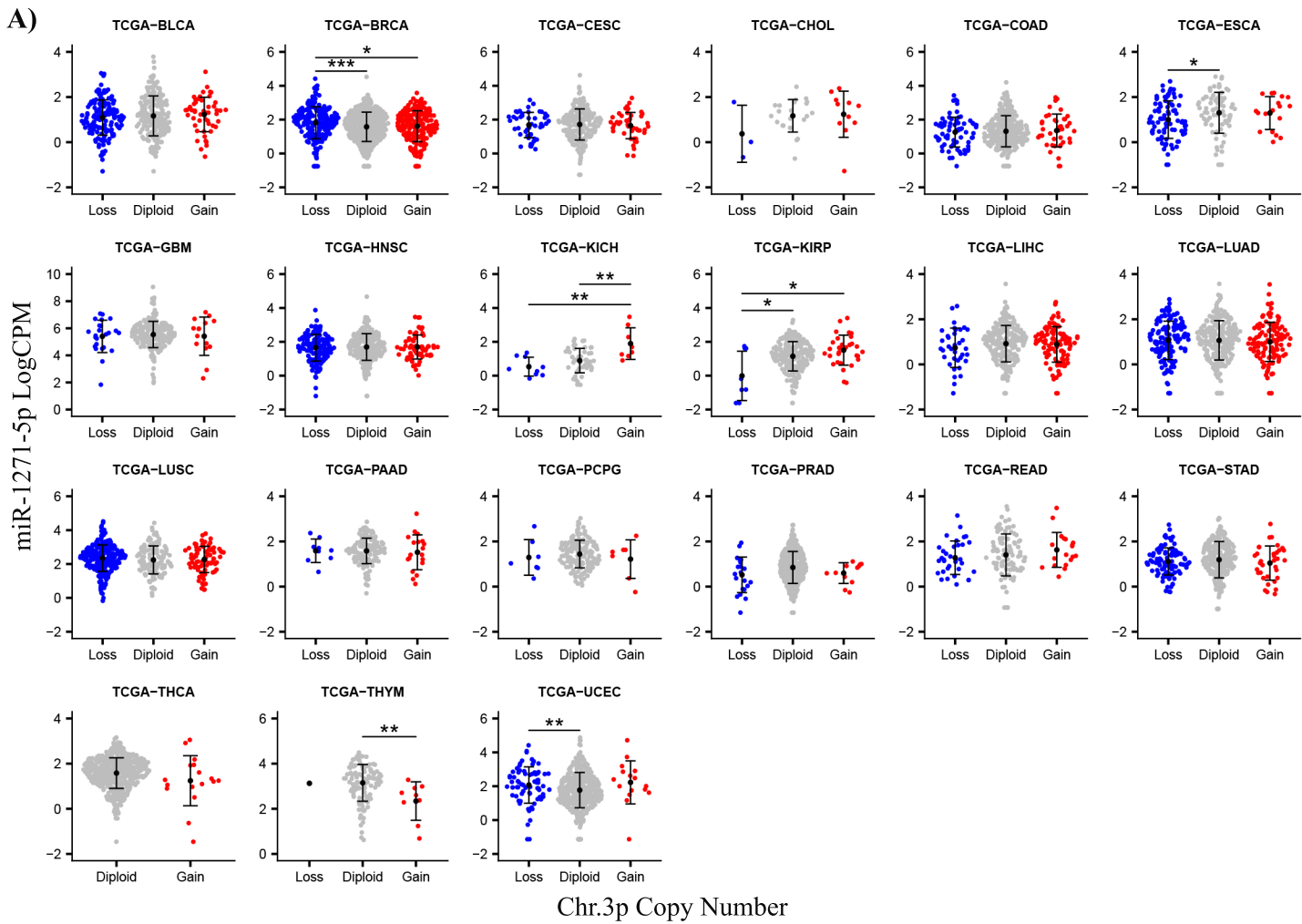

B)

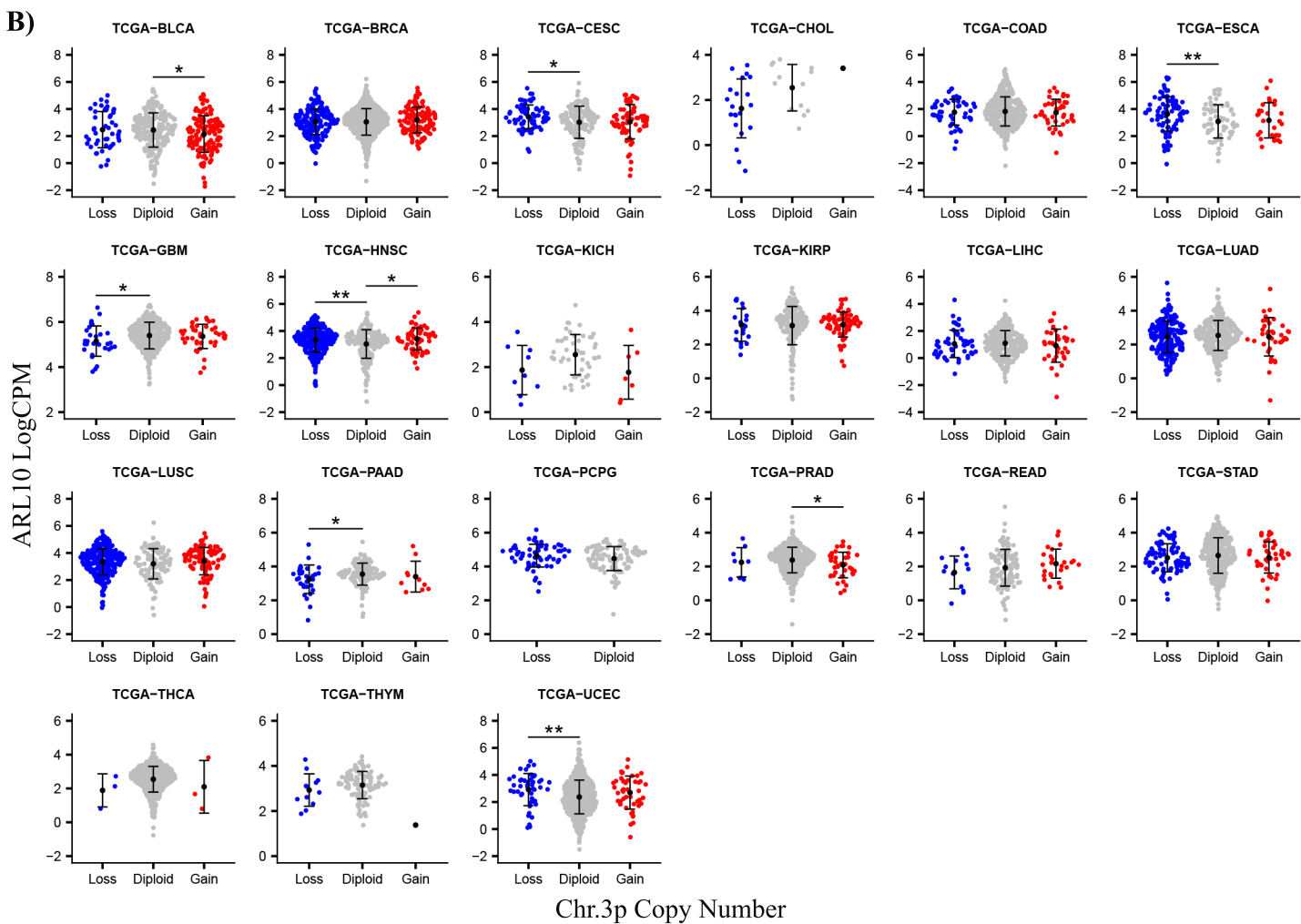

Supplement: Supplementary file 3 — Supplementary Figure 2 [file 41416_2026_3399_MOESM3_ESM.pdf]

**A)**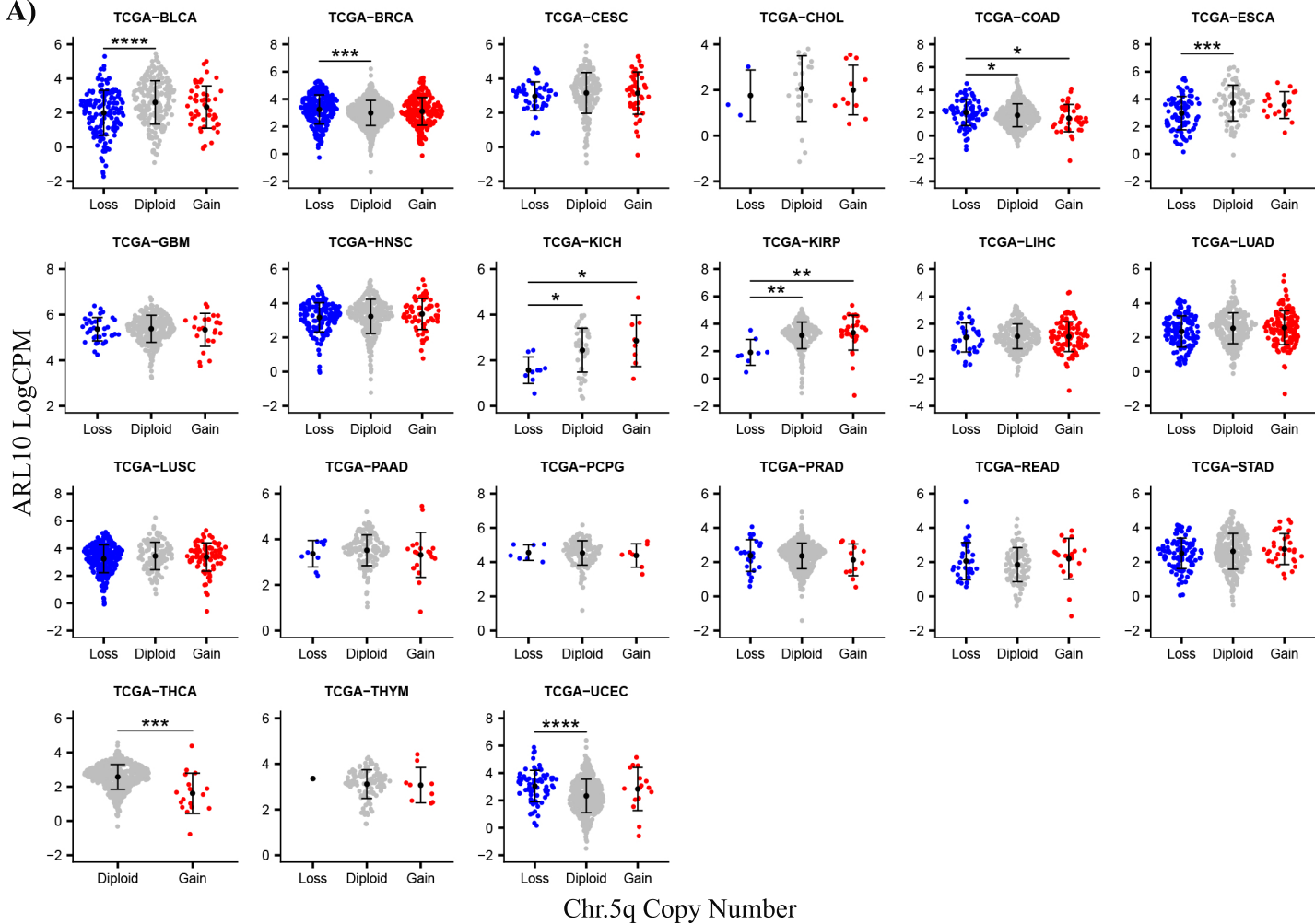**B)**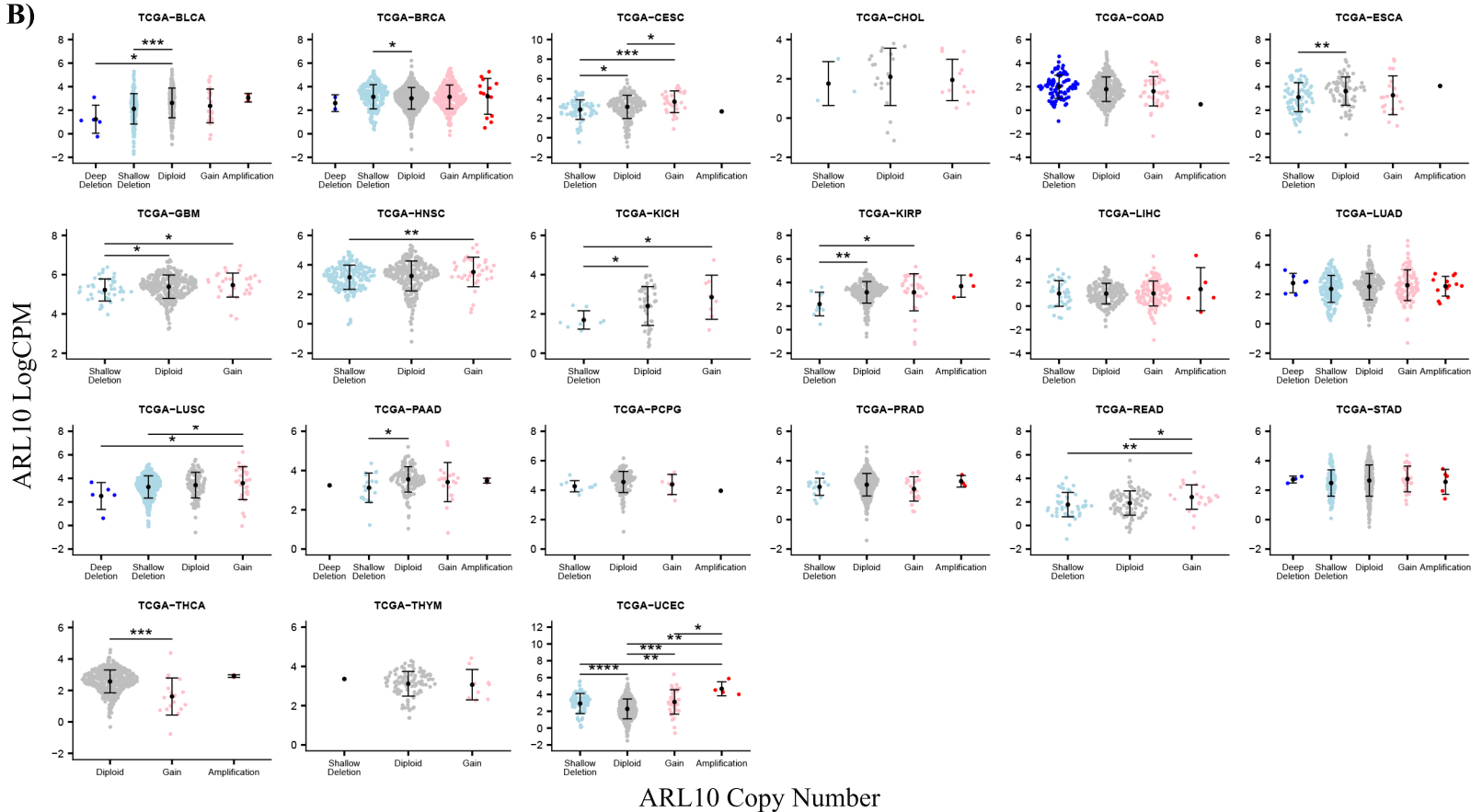

Supplement: Supplementary file 4 — Supplementary Figure 3 [file 41416_2026_3399_MOESM4_ESM.pdf]

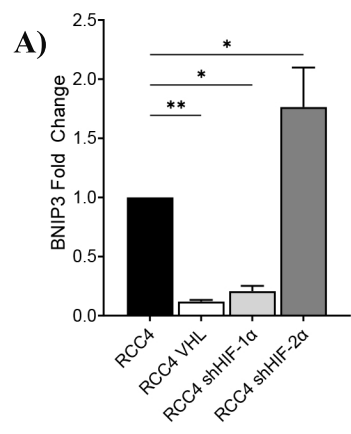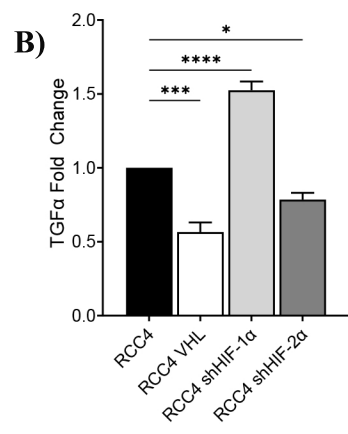

Supplement: Supplementary file 5 — Supplementary Figure 4 [file 41416_2026_3399_MOESM5_ESM.pdf]

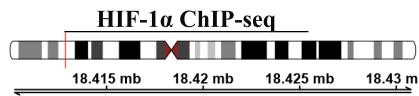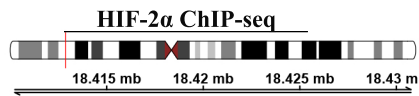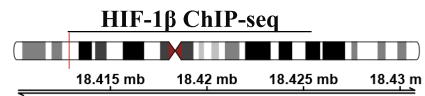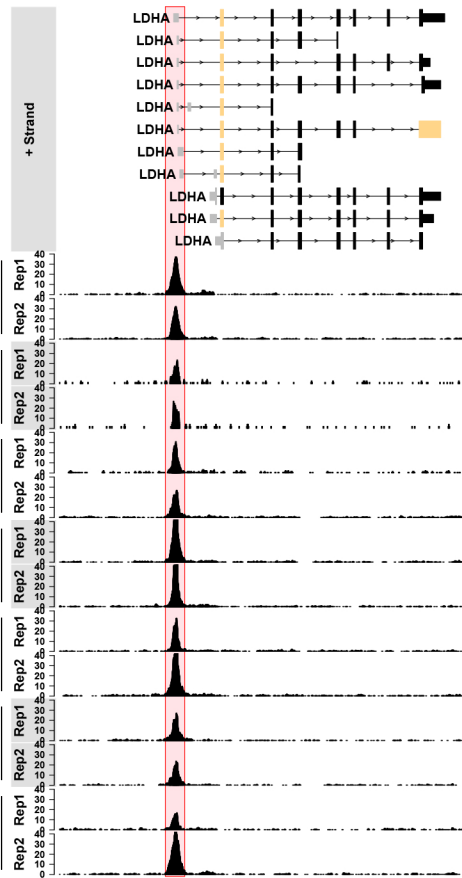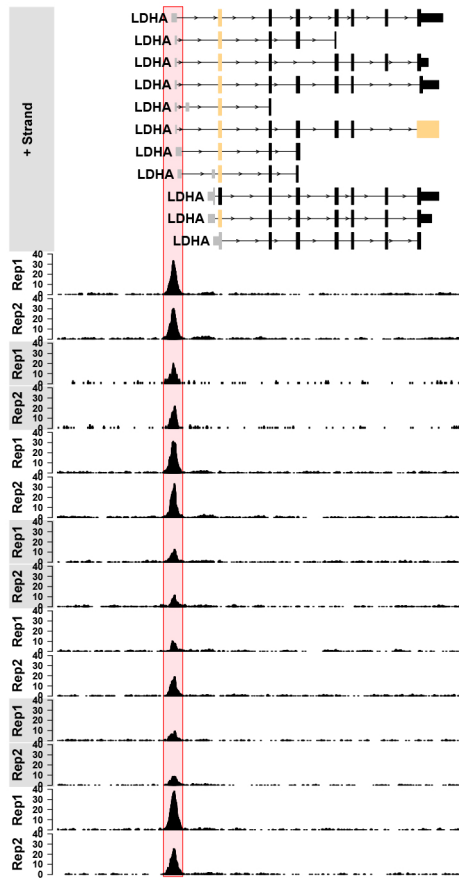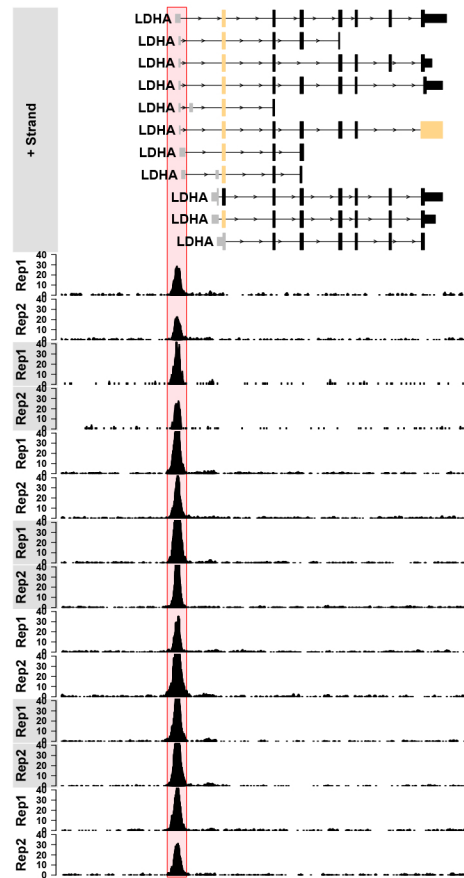

Supplement: Supplementary file 6 — Supplementary Figure 5 [file 41416_2026_3399_MOESM6_ESM.pdf]
